# Defect and strain engineering of monolayer WSe_2_ enables site-controlled single-photon emission up to 150 K

**DOI:** 10.1038/s41467-021-23709-5

**Published:** 2021-06-11

**Authors:** Kamyar Parto, Shaimaa I. Azzam, Kaustav Banerjee, Galan Moody

**Affiliations:** 1 Department of Electrical and Computer Engineering, University of California, Santa Barbara, CA USA; 2 California Nanosystems Institute, University of California Santa Barbara, Santa Barbara, CA USA

**Keywords:** Two-dimensional materials, Quantum optics, Single photons and quantum effects

## Abstract

In recent years, quantum-dot-like single-photon emitters in atomically thin van der Waals materials have become a promising platform for future on-chip scalable quantum light sources with unique advantages over existing technologies, notably the potential for site-specific engineering. However, the required cryogenic temperatures for the functionality of these sources has been an inhibitor of their full potential. Existing methods to create emitters in 2D materials face fundamental challenges in extending the working temperature while maintaining the emitter’s fabrication yield and purity. In this work, we demonstrate a method of creating site-controlled single-photon emitters in atomically thin WSe_2_ with high yield utilizing independent and simultaneous strain engineering via nanoscale stressors and defect engineering via electron-beam irradiation. Many of the emitters exhibit biexciton cascaded emission, single-photon purities above 95%, and working temperatures up to 150 K. This methodology, coupled with possible plasmonic or optical micro-cavity integration, furthers the realization of scalable, room-temperature, and high-quality 2D single- and entangled-photon sources.

## Abstract (web summary)

Quantum defects in 2D semiconductors are promising quantum light sources, but the required cryogenic temperatures limit their applicability. Here, the authors report a method to create single-photon emitters in monolayer WSe_2_ operating at temperatures up to 150 K without plasmonic or optical cavities.

## Introduction

As the second quantum era emerges in the twenty-first century, what were once considered as tangential quantum effects are instead harnessed in new quantum technology. Single-photon emitters (SPEs) are the heart of many quantum photonic applications^1^ such as quantum communication, cryptography, metrology, and linear optical quantum computing. Current state-of-the-art semiconducting single-photon sources^2^—such as InAs self-assembled quantum dots—encounter challenges in scalability and integration with mature photonic technologies due to difficulties in deterministic positioning and growth. In recent years, a new SPE platform^3–8^ has emerged in two-dimensional (2D) materials, including hexagonal boron nitride and transition metal dichalcogenides (TMDs) such as MoSe_2_ and WSe_2_. Monolayer WSe_2_, in comparison to conventional III–V semiconducting quantum dots, offers several advantages, including a high-photon extraction efficiency due to its atomic thickness, the potential for scalable, site-controlled and deterministic manufacturability^9–11^, and ease of integration with mature photonic technologies via simple transfer methods^12^, which has made them an intriguing contender for future technologically relevant quantum light sources.

**Fig. 1:**
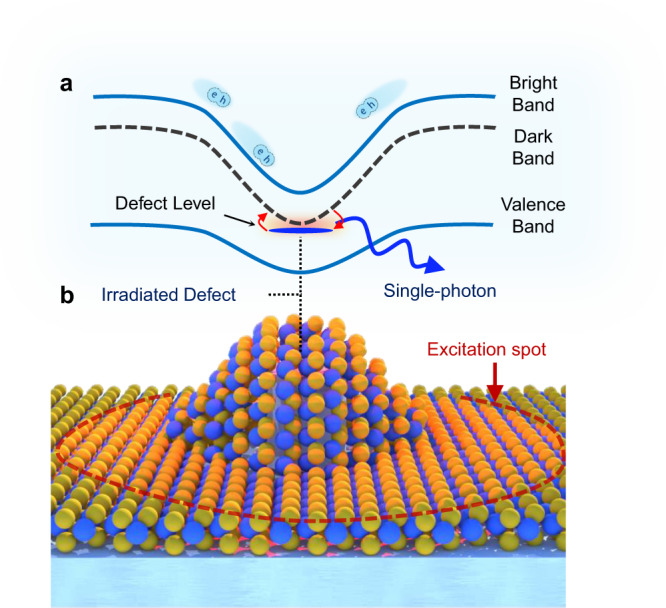
Illustration of a strain and defect-engineered WSe_2_ single-photon emitter. **a** Denotes the spatial bandgap variations due to strain (solid blue lines represent excitonic bright band and valence band, respectively, dashed gray line represents the dark excitonic band). **b** Demonstrates an illustration of 1L-WSe_2_ strained over a SiO_2_ nanopillar (W atoms in blue and Se in yellow). Neutral excitons, represented as e–h pairs, created within the excitation laser spot (red dashed line) funnel to low-potential strained regions. Note that WSe_2_ also has a dark-exciton band at energies below the bright exciton, which is optically forbidden due to the transition selection of WSe_2_. However, at strained regions and in the presence of a defect (top of the irradiated nanopillar, denoted with a blue dash), the dark-exciton can become strain-tuned to a defect-level forming intervalley defect excitons. Given that the defect-level breaks the spin-momentum locking of WSe_2_, the dark-exciton state can then efficiently recombine through the defect level, giving rise to an exponentially bright single-photon emission line (outgoing blue arrow).

However, a challenge facing 2D WSe_2_ SPEs lies in their low working temperature and thermal instability. The ultra-sharp emission lines associated with SPEs that appear ~50–200 meV below the free exciton of WSe_2_ are observable only at cryogenic temperatures and quench above 30 K due to low confinement potential and quantum yield^4,13^. Considering that the microscopic origins of these emitters are still not fully understood, their further optimization and engineering become challenging. Recently, it has been hypothesized that SPEs in WSe_2_ may be attributed to localized intervalley defect-bound excitons that form when the energy of the dark excitonic band of WSe_2_ is lowered and hybridizes with a valley symmetry-breaking defect state at strained regions^14^ (Fig. 1). This description clarifies key points regarding the strong brightness and magneto-optic properties of these emitters, which cannot be justified within an only-strain or an only-defect description. Furthermore, it reveals that both strain and defects are pivotal in the creation of SPE. Recent experimental studies^15,16^ have also confirmed parts of this model, however, the essential link, which is the correlation between defects and quantum emitters, remains elusive. This is partially because defects in 2D TMDs are ubiquitous and difficult to control. The challenge compounds further since most strain engineering methods are also prone to damage or pierce the TMDs in the process^9^.

In this study, by using electron-beam (e-beam) irradiation as a controllable method to induce structural defects in WSe_2_, along with engineering strain fields in encapsulated WSe_2_ using dielectric nanopillar structures, we decouple the strain and defect engineering processes and prove that there is a direct correlation between defect density and quantum emitters, which provides strong evidence for the intervalley defect exciton model. Moreover, the accurate positioning of defects within the strain field in WSe_2_ minimizes the non-radiative recombination and achieves higher confinement potential. These, in addition to the possible distinct morphology of our e-beam engineered defects that appear at deeper energies than any other observed SPEs in WSe_2_, enable engineering SPEs with high yield, high single-photon purity, biexciton-like radiative cascade, and possible working temperatures up to 215 K. This sets the precedent for the highest working temperature of SPEs observed in 2D TMDs without Purcell enhancement (benchmark available in Supplementary Note 1).

## Results

### Strained WSe_2_ PL response

**Fig. 2:**
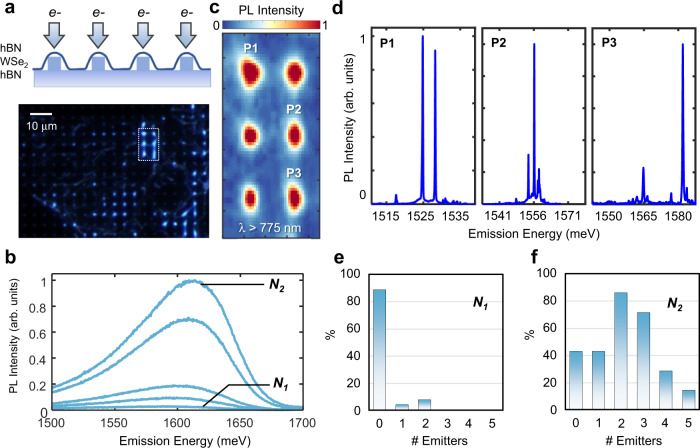
Site-controlled quantum emitter array **via** strain and defect engineering. **a** Schematic of the fabricated structure and the optical dark-field image of the sample. An stack of h-BN/WSe_2_/h-BN is transferred on a SiO_2_ nanopillar array. Bright emission is observed from the top of the pillars due to exciton funneling. Next, top of the pillars are irradiated (e^−^ arrows) to induce structural defects. **b** PL spectra of unstrained WSe_2_ defect-bound excitons as a function of irradiation intensity from *N*_1_ = 10^5^ electrons/μm to *N*_2_ = 10^6^ electrons/μm 10 μW excitation power. **c** Spectrally integrated PL map of strained and irradiated sites for wavelengths above 775 nm, showing the bright emission associated with single-photon emitters. Some sites host more than one emitter and have reached saturation incident count. The maps resemble the Gaussian spot size of excitation laser due to the localized nature of the emitters. **d** PL spectrum (1 μW excitation power) captured from different pillar sites showing the sharp emission lines (75 µeV average linewidth) associated with SPEs. Note that many of the sharp emission lines appear in pairs, which indicates the possible formation of exciton-biexciton pairs. **e** Histogram of number of quantum emitters measured per site by irradiating with e-beam intensity of 10^5^ electrons/μm^2^. **f** Histogram of number of quantum emitters measured per site for 10^6^ electrons/μm^2^.

A monolayer WSe_2_ sample encapsulated by h-BN was prepared using dry transfer techniques (see Methods). h-BN flakes, with thickness of less than 5 nm, were chosen to encapsulate the WSe_2_ not only to isolate the WSe_2_ from surface states and adsorbates^17^, but to also act as protective layers that shield the WSe_2_ from potential damages during the stamping process onto nanopillars. Subsequently, to engineer the necessary strain profile across the 2D flake, the stack was transferred onto a Si/SiO_2_ substrate array with a predefined nanopillar dielectric array with height and diameter of 200 and 150 nm, respectively, which was previously identified as an optimal aspect ratio for deterministically introducing SPEs without piercing through the materials^10^. Figure 2a presents the dark-field optical image of the final assembled structure where the brightly illuminated regions correspond to the nanopillar sites. Photoluminescence (PL) spectra of the WSe_2_ residing on the nanopillars were taken at *T* = 5 K (Supplementary Fig. 1). Interestingly, no sharp emission peaks that could be associated with single-photon emitters were observed. In contrast, only slightly redshifted neutral exciton (X) and charged exciton (X^−^) peaks were detected. Given that the strained region is about 2-orders of magnitude smaller than the diffraction-limited spot size, the red-shifted neutral exciton peaks due to strain are dim and cannot be resolved by a far-field diffraction-limited system (see Supplementary Note 2 and Supplementary Figs. 2 and 3), consistent with previous studies^18,19^. Furthermore, the defect band that usually dominates the PL spectra of 1L-WSe_2_ is also absent in the PL spectrum both in the strained and unstrained regions for the typical excitation powers and accumulation times used in our experiments. However, a very weak broad-defect band, without any sharp peaks, can be observed when PL intensity at low excitation power is integrated over a long time. Interestingly, the correlation between the defect band and quantum emitters has been hinted in previous studies where the spectral histogram of measured emitters reconstruct the shape of the defect band^9,11^. Overall, this provides evidence that in WSe_2_ samples, which are devoid of the defect band and are shielded from the substrate with h-BN encapsulation, single-photon emitters cannot form solely due to the strain in WSe_2_.

### PL response of defect-induced irradiated WSe_2_

To further test this hypothesis and examine correlations between defects and quantum emitters, electron-beam irradiation, which has been successfully used to induce structural defects^20,21^ and phase transformation^22^ in 2D TMDs, was utilized to induce defects at desired locations. First, an unstrained region away from the nanopillars was irradiated at five different intensities ranging from 8 × 10^4^ electrons/μm^2^ to 10^6^ electrons/μm^2^ with an accelerating voltage of 100 keV and an e-beam spot size that is tightly focused (<10 nm) and is held constant to maximize the spatial accuracy. Figure 2b shows the PL spectra of unstrained regions after e-beam irradiation at the energy range corresponding to the WSe_2_ defect band. At low irradiation intensity (<10^5^ electrons/μm^2^), the band is very dim and difficult to distinguish. However, as the irradiation intensity increases, the defect band intensifies, which indicates that e-beam irradiation generates optically active defects in WSe_2._ These observations are consistent with previous studies^21^ and it should be emphasized that the broad defect band emission retains its qualitative characteristics irrespective of the power used^21^_._ It is readily observable that solely with e-beam irradiation, only the defect band can be created, and no sharp quantum emission lines appear in the spectra.

### Strain and defect-engineered quantum emitters in WSe_2_

Next, we irradiated the strained regions at the top of the nanopillars as shown in Fig. 2a with the same nominal intensities. At the lowest intensity, no quantum emitters were observed in our samples. However, at the threshold of 10^5^ electrons/μm^2^ (denoted as *N*_1_), 12% of sites demonstrated at least one quantum emitter (Fig. 2e). As the electron irradiation intensity increases further, both the yield and number of emitters per site increase. At 10^6^ electrons/μm^2^ (*N*_2_), 85% of the emitters demonstrated at least one quantum emitter per site (Fig. 2f). It is worth noting that e-beam irradiation has also been utilized to generate SPEs in h-BN, however, the h-BN emitters are not enabled unless a thermal annealing step is performed. Considering our cryogenic temperature and the fact that emitters also appear at lower energies compared to h-BN emitters at room temperature, the emitters studied here are highly unlikely to originate from h-BN. Lastly, a recent study^23^ has successfully used helium ion bombardment to create defects exhibiting single-photon emission in 2D MoS_2_, however, we must emphasize here that WSe_2_ due to its dark ground-state, is a phenomenological interesting case wherein inducing defects through irradiation alone will not result in SPE creation. Integrated PL maps (Fig. 2b) of six irradiated nanopillars (white dashed box region in Fig. 2a were taken at 5 K for energies below 1.6 eV. These maps provide evidence for the formation of bright emission lines below the WSe_2_ free exciton. The correlation among e-beam intensity, optical emission intensity of the defect band, and the number of engineered quantum emitters in each site strongly supports the hypothesis that both strain and defects have a fundamental role in single-photon emission in WSe_2_^14–16^.

Figure 2d shows the PL spectrum at the location of three different irradiated nanopillars. Sharp emission lines associated with SPEs in these materials appear at the energy interval of 1515–1580 meV. Note that this energy interval is approximately 100 meV lower than the usual energy range^3–7,9–12^ that SPEs appear in WSe_2_. This suggests that the morphology of the e-beam-induced defects may be distinct from the previously observed native defects and reside deeper in the bandgap.

### Observation of biexciton emission cascade

**Fig. 3:**
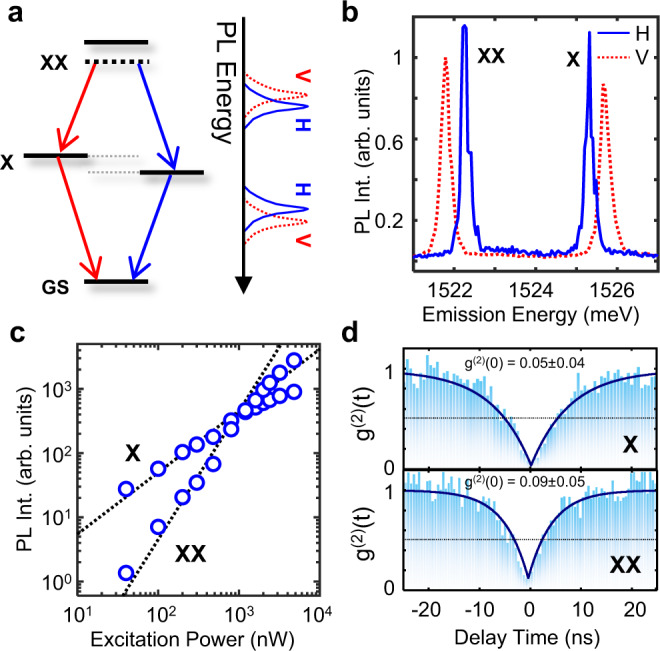
Single-emitter photoluminescence and photon antibunching. **a** Schematic of a cascaded biexciton emission proces. GS, X, and XX denotes the ground-state, exciton, and the biexciton states respectively. Biexciton first decays to an exciton either by emitting a vertically (V) or horizontally (H) polarized photon denoted by red or blue arrows respectively. Subsequently, the exciton decays to ground-state by emitting a second vertically or horizontally polarized photon with polarization correlated to the initial emitted photon, completing the emission cascade. The energy splitting between the different linear polarization arises due to the fine-structure splitting. **b** PL Spectrum of a single-quantum emitter. Two pairs of correlated doublets are observed. **c** PL intensity vs. Excitation Power: The localized defect exciton shows distinct behavior: Exciton (X) intensity increases sublinearly and begins to saturate at high powers as expected from a two-level system, whereas the biexciton line (XX) intensity increases superlinearly. Dashed lines are linear fit (log (PL Int) = x log (Excitation Power)) to the data with extracted slopes of 0.96 for exciton (X) and 1.587 for biexciton (XX), respectively. **d** Second-order correlation measurement. Both X and XX exhibit *g*^(2)^(0) < 0.1, showing high-purity single-photon emission.

With a closer look at Fig. 2d, it is readily observable that many sharp emission lines appear to form in pairs with an energy spacing of about 3–5 meV. The features of these peaks resemble those of an exciton-biexciton pair in which the inherent fine-structure splitting results in two doublets, but the sign of the splitting is reversed in each pair as a consequence of the emission cascade (Fig. 3a). The polarization-resolved PL spectrum of the pairs further corroborates the radiative cascade (Fig. 3b). The integrated intensities of the pairs show distinct sub-linear and super-linear characteristics as previously observed for exciton-biexciton emissions in WSe_2_^24^ (Fig. 3c). Similarly, the time-resolved PL (Supplementary Fig. 4) also shows that the decay time (T_1_) of the exciton (X, T_1_ = 6.12 ns) feature is almost 1.5 times higher than the biexciton (XX, T_1_ = 4.01 ns), which is comparable with the previously observed dynamics of biexciton-exciton pairs in WSe_2_^24^. Note that the physical origin of these exciton-biexciton-like features is still ambiguous; previous studies^14^ have shown that such features can also be attributed to the hybridization of defect states with localized excitons. Spectral diffusion of both exciton and biexciton was measured in a 5-min period, which showed almost appreciable spectral wandering (Supplementary Fig. 5). Finally, second-order correlation measurements were performed using a Hanbury Brown and Twiss setup^25^ that confirms both emission lines act as single-photon sources with purities as high as 90% (Fig. 3d).

### Statistics of the defect and strain engineered sites

**Fig. 4:**
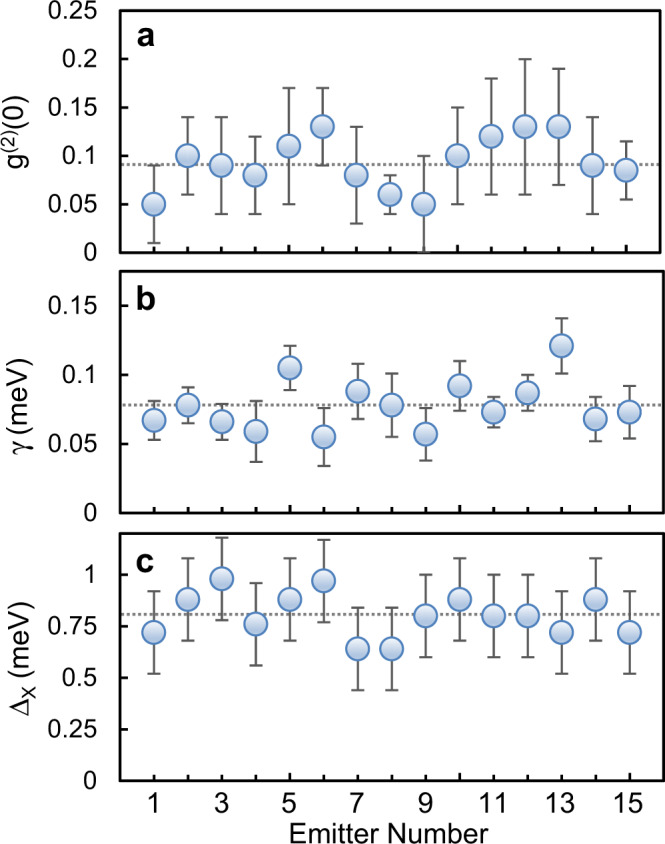
Statistics of single-emitter properties. **a** *g*^(2)^(0) distribution. Average *g*^(2)^(0) is 0.08, which demonstrates the high purities achieved by this method. **b** Homogenous linewidth distribution with 75 μeV average. **c** Zero-field doublet-splitting showing the reproducibility of the method in engineering SPEs with similar characteristics. Horizontal dashed lines denote the average of measurements. Error bars denote the standard deviation from fitting the data.

Engineering high purity and reproducible single-photon emitters with high yield is a requirement for any scalable photonic technology. By decoupling the strain and defect engineering in our nanopillar irradiation process, we were able to achieve a success rate of over 85% in engineering single-photon emitters per site. Note that, since e-beam irradiation is a repeatable process, subsequent irradiation and dosage optimization processes can be leveraged to achieve near-unity-yield at each site. Figure 4 represents the statistical histograms of the single-photon emitters fabricated using our method. The spectral purity of many of the emitters was measured to be above 95% (with an average purity of 92%, Fig. 4a) with average linewidths of 75 μeV and zero-field splitting of 760 μeV, and the linewidth and purities will likely improve with resonance excitation.

Furthermore, the energy interval of the observed SPE emission (1515–1580 meV) is smaller than other approaches. This is likely a feature of our decoupled approach—first a broad-defect band is engineered with e-beam irradiation (~1.5–1.7 eV in Fig. 2). The SPEs then appear when a localized stressor red-shifts the neutral exciton energy into resonance with a defect state in the band. Given the defect band range (1.5–1.7 eV) is larger than the spectral range of the emitters (1515–1580 meV), it can be deduced that the variance in the engineered strain profiles sets the limit for the spectral spread of our method. In future experiments, using more precise strain engineering methods may enable engineering identical vdW SPEs.

### Temperature evolution of engineered WSe_2_ emitters

As discussed previously, one of the main disadvantages of native WSe_2_ single-photon emitters is that their PL intensity and single-photon purity quench at temperatures above 30 K. One of the main contributors to this effect is the defect-bound exciton–phonon interaction, which leads to carrier escape from the confined potential, an effect which is more dominant in shallow quantum emitters. As e-beam-induced defects create deeper states within the bandgap, as evidence by the lower emission energies compared to native defect-based emitters, a higher energy barrier and hence, a higher thermal activation energy is to be expected^26^. In addition to this, the non-radiative trap-assisted recombination can become the dominant quenching factor at high-trap densities^27^. In our process the weights of non-radiative recombination pathways are minimized by using exfoliated 2D WSe_2_ with low-defect density (evident from the absence of the defect band). Conversely, the defects are induced in the sample via a controllable process targeting only the desired strained regions. Hence, our decoupled approach also alleviates this mechanism and enhances the quantum yield of the emitters to further increase the working temperatures. It is worth mentioning that higher confinement potentials can be achieved through more accurate positioning of the defect site within the strain field.

**Fig. 5:**
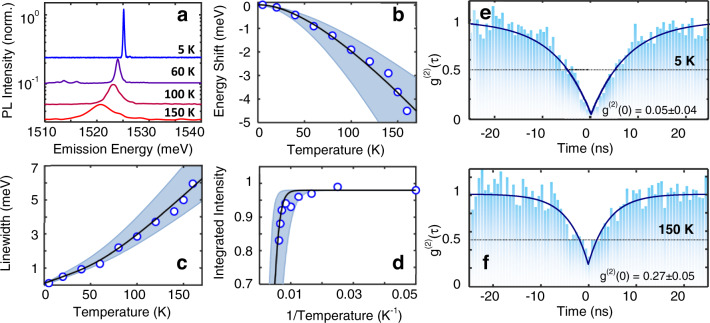
Statistics of single-photon emission up to 150 K. **a** Evolution of the PL spectrum as function of the temperature. **b** Redshift of the SPE line with temperature increase due to the reduction of the bandgap following Varshni’s empirical relationship. **c** Homogenous linewidth broadening due to the increase of temperature. **d** Arhenious plot of the integrated intensity. The PL peak starts to quench around 150 K and reaches e^−1^ at 215 K. Shaded region in **b**, **c**, and **d** demonstrate the range of recorded parameters for an ensemble of emitters. Open circles demonstrate measured data for a single emitter in the ensemble and the solid black lines are theoretical fit to the data. **e** Second-order correlation measurement *g*^(2)^ at *T* = 5 K and **f** at 150 K exhibiting *g*^2^(0) < 0.3, evidencing that the single-photon nature persists up to our maximum measurement temperature of 150 K and likely persists beyond 200 K. Horizontal dashed lines represents the quantum limit of *g*^(2)^ at 0.5.

Figure 5a shows the evolution of the single-photon PL emission from 5 to 150 K (Supplementary Note 3 and Supplementary Fig. 6 are dedicated to the quantitative analysis of single-emitter properties). It is readily apparent that the emission line is distinguishable up to 150 K. A ~5 meV redshift is distinguishable up to 150 K (Fig. 5b), which indicates that deep confinement decouples the exciton energy from the temperature dependence of the lattice. Fitting the temperature dependence (Fig. 5b) to a Varshni-form equation (Supplementary Note 3) indicates a temperature coefficient (5.3 × 10^−6^ eV K^−1^), which is smaller than the previously reported values for native defects in CVD grown TMDs^13^. The evolution of the homogenous linewidth broadening with temperature is indicative of a thermally activated pure dephasing from exciton–phonon scattering and can be seen in Fig. 5c. The Arrhenius plot of the integrated intensity of the SPE vs. temperature shows that the integrated intensity quenches to e^−1^ at ~215 K (Fig. 5d). The data are fit with an Arrhenius model *I*(*T*) = *I*_*0*_/(1 + *R* exp⁡(−*E*_*A*_/*kT*)), where *R* is equal to the ratio of the radiative (*T*_*r*_) and non-radiative (*T*_*nr*_) recombination lifetimes and *E*_*A*_ is the thermal activation energy that required to dissociate the defect-bound excitons. The fit to the data results in a quantum yield of 5% and an activation energy of 95 meV, suggesting that the higher activation energies observed in this work is the main factor that contributes to high working temperatures of our emitters (see Supplementary Fig. 6 for a comparative analysis). It is worth mentioning that linewidth narrowing and an increase in intensity caused by h-BN encapsulation also contribute to higher working temperatures. However, given that the phonon escape quenching mechanism exponentially affects the PL intensity, whereas linewidth broadening, and initial intensity (*I*_0_) have a linear dependence, this enhancement is limited. This is further corroborated by the previous demonstrations of h-BN encapsulated TMD defect-bound excitons where overall a limited enhancement of working temperatures are observed^23,28^, but the fundamental quenching mechanism is not altered by h-BN encapsulation.

Figure 5e, f show the temperature dependence of the second-order correlation function and demonstrates that the emitters exhibit antibunching up to 150 K with *g*^(2)^(0) = 0.27 ± 0.05. Given the high average purity (~75%) of the array of emitters at 150 K (see Supplementary Fig. 7) in combination with the Arrhenius data, it can be expected that the single-photon nature of the SPEs can persist up to 215 K. It can be predicted that our SPE engineering method, in tandem with designs leveraging Purcell enhancement, could enable room temperature single-photon emission.

## Discussion

**Fig. 6:**
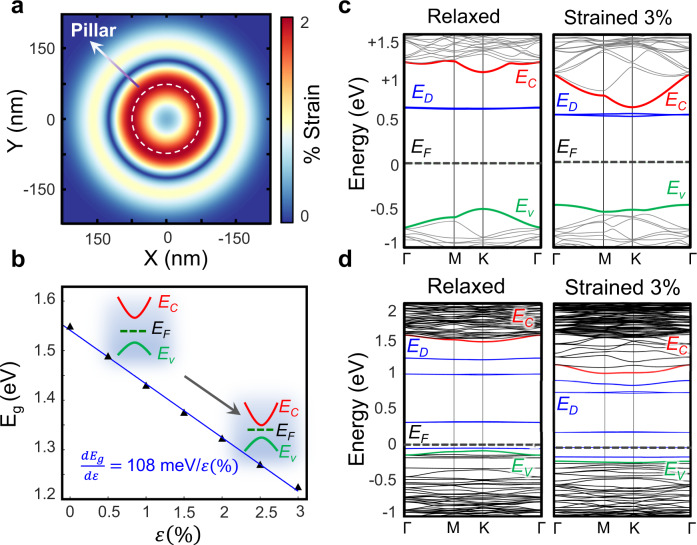
DFT Calculations of strained defect bandstructure. **a** Simulated strain profile at the nanopillar location. **b** Bandgap as a function of applied biaxial tensile strain. The inset band diagram denotes the qualitative movements of conduction band (*E*_C_), Valence band (*E*_V_), and the Fermi-level (*E*_F_) with strain. Note that the conduction band movement is larger with respect to the valence band. **c** Evolution of the band diagram of selenium vacancy with strain. *E*_D_ and respective blue bands denote the localized defect levels. Upon the application of strain, *E*_c_ and *E*_D_ come into proximity. **d** Evolution of the band diagram of WSe_6_ pore complex vs. strain. After the application of strain, the energy spacing between the conduction band and its closest defect-level decreases. Note that the Fermi-level energy position depends on the size of the supercell.

Figure 6 quantitatively summarizes one plausible mechanism behind the single-photon emission as depicted in Fig. 1. The dielectric nanopillar creates a strain profile akin to Fig. 6a in the 2D flake where the strain is at its maximum around the edges of the nanopillar. The applied strain results in the reduction of the bandgap (Fig. 6b), which creates a potential landscape analogous to Fig. 6a. This potential leads to the localization of free exciton states. From e-beam irradiation, defect states appear within the bandgap of the semiconductor in the strained regions. Provided the energy of these defect states at the strained regions is sufficiently close to the localized exciton states, the defects and excitons hybridize and results in the bright single-photon emission peak. While the engineering methods outlined in this work demonstrate the interplay between defects and strain, as predicted by the above-mentioned picture, the physical morphology of the defects responsible for SPE remains elusive. There is a lack of consensus of the defects responsible for single-photon emission, but they have been previously attributed to structures, including selenium vacancies^14^, tungsten centered vacancies^29^, oxygen interstitials^30^, and anti-site defects^31^. It is also plausible that multiple types of defects may be responsible for single-photon emission as long as they break the valley symmetry, and upon application of strain, introduce defect states with favorable energies close to the conduction band.

Given that the defects in this work were created by e-beam irradiation for which the morphology of resulting defects has been extensively studied, the list of possible suspects can be reduced through a systematic, ab initio approach. Studies have shown that e-beam irradiation processes primarily contribute to the generation of chalcogen and double chalcogen vacancies due to a lower knock-off energy^20^. Furthermore, prolonged exposure of a focused e-beam spot can also lead to the destabilization of the transition metal bond, which causes the transition metal to migrate away from the site, creating more complex vacancy sites such as pore vacancies^32,33^. In the case of WSe_2_, the creation of rotational trefoil defect complexes under e-beam irradiation has also been observed^34^. Therefore, we chose to study five probable defect complexes that might form during the e-beam process, namely selenium single vacancy, selenium double vacancy, tungsten vacancy, pore vacancy, and trefoil vacancies (see Supplementary Note 4 and Supplementary Figs. 8–13 for full details).

Density-functional theory (DFT) calculations were performed using Synopsis QuantumATK^35^ package by incorporating Perdew–Burke–Ernzerh (PBE) variant of generalized gradient approximation^36^ (PBE-GGA) exchange-correlation functional (see Methods for details). Note that given the common bandgap underestimation problem in DFT, along with the uncertainties in the exciton binding energies and the magnitude of the confinement potential, our focus is not on a quantitative comparison. Instead, our main criterion is the relative movement of the conduction band and defect levels with respect to each other upon application of strain, which can be assessed using ab initio simulation.

Figure 6c shows the dynamics of selenium single-vacancy levels with applied biaxial tensile strain. It is readily observable that the defect levels appear at favorable energies close to the conduction band and move to the proximity of the conduction band with strain. However, both selenium vacancies and selenium double vacancies are prone to passivation by oxidation^37^. Given the fact that the SPEs in this work, similar to previous studies, remain functional after many days and temperature cycles, these defects are an unlikely candidate. Conversely, tungsten vacancies do not introduce energies in the vicinity of the conduction band and, therefore, are not energetically favorable (see Supplementary Fig. 11). WSe_6_ vacancy complex—pore vacancies—on the other hand, form nanopores similar to those observed in MoS_2_^32^ and WS_2_^33^ after geometry relaxation (Supplementary Figs. 12 and 13). These vacancies also introduce energies close to the conduction band and move closer upon further application of strain (Fig. 5d). Thus, it is plausible that more complex vacancy structures created by e-beam irradiation, such as nanopores, are responsible for the SPEs observed in the e-beam irradiated sites.

In conclusion, by decoupling the strain and defect engineering process in the design of WSe_2_ single-photon emitters, we were able to achieve near-unity yield in deterministic positioning of quantum emitters with high purity that can preserve their single-photon nature to well above 150 K. The result indicates that both defects and strain are fundamental to quantum emitters in our WSe_2_ samples. This method was also successful at deterministic engineering of exciton-biexciton-like features with a radiative cascade that may pave the way for the realization of entangled photon-pair sources. It can be expected that by utilizing the methodology described in this study and integration with plasmonic or microphotonic cavities, our work may set the stage for future scalable elevated-temperature 2D WSe_2_ quantum light sources.

## Methods

### Sample preparation

The nanopillar array was fabricated with high-resolution electron-beam lithography following the procedures described in ref. ^10^. Briefly, a silicon wafer with thermal oxide was cleaved into a 1 cm^2^ chip followed by a solvent clean. The chip was spin-coated with hydrogen silsesquioxane (HSQ) resist diluted with methyl isobutyl ketone (MIBK). After a 5-min bake at 90 °C, pillar arrays and chip alignment markers were defined via electron-beam lithography and then developed in a 25% tetramethyl ammonium hydroxide (TMAH) solution and rinsed with methanol. A final rapid thermal anneal step at 1000 °C in oxygen converts the HSQ pattern into SiO_2_. The nanopillars have a nominal diameter of 150 nm and height of 200 nm.

We used a dry viscoelastic transfer technique to create the h-BN-encapsulated WSe_2_ structure. Intrinsic WSe_2_ flakes (2D Semiconductors) were exfoliated onto an unpatterned preparation SiO_2_/silicon substrate for identification and subsequent transfer. Next a thin flake of h-BN is exfoliated onto the transfer stamp, which was used to exfoliate and transfer WSe_2_ monolayers from thicker flakes on the preparation substrate. Finally, the WSe_2_/h-BN stack was transferred to an h-BN layer exfoliated on the patterned nanopillar array, which ensures that the WSe_2_ layer is encapsulated by the h-BN and free of any contaminants or residues during and after the transfer process. Quantum emitters were created using a 100 keV, <10 nm spot size electron-beam to expose the sample at the location of each nanopillar with ~10^6^ electrons/μm^2^.

### Optical spectroscopy

For steady-state micro-photoluminescence (PL) measurements, the sample temperature was held fixed between 5 and 150 K. The emitters were excited non-resonantly with a continuous-wave 633 nm laser focused to a ~1 μm full-width at half-maximum spot size using a long working distance 0.7 numerical aperture microscope objective. The backscattered PL was collected and measured with a spectrometer and TE-cooled charge-coupled device with a spectral resolution of ~30 μeV. For time-resolved PL measurements, a 40 MHz, 635 nm pulsed laser diode was used as the excitation source. The PL was spectrally filtered with ~1 nm bandwidth and detected using a superconducting nanowire single-photon detector and time-tagging electronics with ~40 ps detector temporal resolution. The PL measurements were performed at various average excitation powers ranging from 10 nW to 10 μW. Polarization-resolved spectra were acquired with a half-wave plate and linear polarizer before the spectrometer.

### DFT calculations

HGH pseudopotentials with Tier4 basis sets, 3 × 3 × 1 and 3 × 3 × 1 Brillouin zone k-point sampling, 200 Rydberg density mesh cutoffs and a 0.02 eV/Å maximum force constant were used for geometry optimizations and DFT calculations. GGA-HGH Tier4 setup has been widely used in literature to probe the physics of 2D TMDs. WSe_2_ supercell was geometrically relaxed using the setup discussed above to 0.02 eV/Å maximum force constant. Next, depending on the size of the defect complex, a 5 × 5 or 7 × 7 supercell was constructed, followed by the removal of the appropriate atoms. Next, the geometry was optimized again by constraining the supercell along the in-plane direction to achieve the target strain and allowing for the defective supercell to relax to its final configuration.

## Supplementary information

Supplementary Information

## Data Availability

The data in this manuscript is available upon reasonable request.
