# Supplementary material for: Defect and strain engineering of monolayer WSe2 enables site-controlled single-photon emission up to 150 K
Source: Nat Commun. 2021 Jun 11;12:3585. doi: 10.1038/s41467-021-23709-5 (PMC8196156; doi:10.1038/s41467-021-23709-5)
Supplement: Supplementary file 1 — Supplementary Information [file 41467_2021_23709_MOESM1_ESM.pdf]

Supplementary Information

Defect and Strain Engineering of Monolayer WSe_2 Enables Site-Controlled Single-Photon Emission up to 150 K

Kamyar Parto[1], Shaimaa I. Azzam[1,2], Kaustav Banerjee[1,2], and Galan Moody[1,2*]

[1] Electrical and Computer Engineering Department, University of California Santa Barbara, Santa Barbara, CA 93106
[2] California Nanosystems Institute, University of California Santa Barbara, CA 93106

*moody@ucsb.edu

- **Supplementary Note 1**
  WSe_2 Single-photon emitter temperature benchmark
- **Supplementary Figure 1**
  Photoluminescence of strained (top of the pillar) and unstrained WSe_2 without e-beam irradiation
- **Supplementary Note 2, Supplementary Figure 2-3**
  Predicted PL-spectra of strained WSe_2 (top of the pillar) including diffraction-limited finite-spot size correction
- **Supplementary Figure 4**
  Time-resolve PL
- **Supplementary Figure 5**
  Spectral Diffusion of Exciton (X) and Biexciton (XX)
- **Supplementary Note 3, Supplementary Figure 6**
  Temperature dependence of the single emitters
- **Supplementary Figure 7**
  Statistics of single emitters across the ensemble
- **Supplementary Note 4, Supplementary Figure 8-13**
  DFT simulation details

Supplementary Note 1: Benchmark of the single-photon demonstrations in WSe_2:

Note some of the following studies did not intend to study thermal behaviour of the SPE emitters and hence, the maximum reported temperature may not reflect the actual maximum temperature of the emitters.

Name of Study	Purity	Maximum reported working temperature	Yield	Comments
**Srivastava et al [1]**	**78-80 %**	**4.2 K**	×	
**Chakraborty et al [2]**	**64 %**	**35 K**	×	
**Kumar et al[3]**	**83 %**	**4 K**	×	
**Tonndorf et al[4]**	**68-73 %**	**10 K**	×	
**He et al[5]**	**79-86 %**	**14 K**	×	
**Kern et al[6]**	**77-81 %**	**10 K**	Semi-deterministic	
**He et al[7]**	**61-78 %**	**4.5 K**	×	
**Branny et al[8]**	**93-97* %**	**3.5 K**	96%	Average purity not reported.
**Palacios et al[9]**	**82-92%**	**10 K**	95* %	* ~63% including the pierced sites.
**Luo et al[10]**	**78-84%**	**3.8 K**	95* %	* Each site contains 4 possible locations for single-photon emitters. The success rate for engineering emitters per location reported at 67%
**Rosenberger et al[11]**	**59-57%**	**60 K**	Deterministic - yield not reported	
**Kim et al[12]**	**78%**	**4 K**	Deterministic - yield not reported	
**Luo[13]**	**76-80%**	**60 K* (Without Purcell enhancement)**	Similar to [10]	* 160 K was achieved using Purcell enhancement.
**This work**	**88-95% (92% avg)**	**150 K (Possibly 215 k)**	85%	

Supplementary Figure 1: Photoluminscence of strained (top of the pillar) and unstrained WSe_2 without e-beam irradiation:

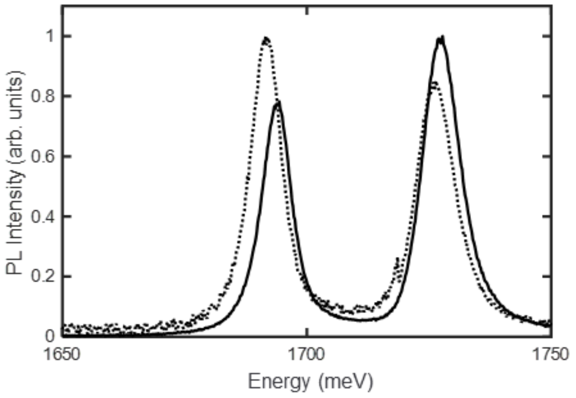

Supplementary Figure 1: PL of neutral and charged excitons on un-irradiated encapsulated WSe_2. Dashed curve presents the PL from top of the nano-pillars (strained regions) and solid curve denotes the PL away from nano-pillars (unstrained regions). On nano-pillar about 1.8 meV (exciton) and 3.1 meV (charged exciton) is observed. Data normalized to maximum intensity for each curve. This redshift was also within the uncertainty of sample-to-sample variations. The linewidth of the neutral exciton away from the pillar is 8 meV and does not show any appreciable changes compared to linewidth of the exciton taken from the pillar site.

Supplementary Note 2: Predicted PL-spectra of strained WSe_2 (top of the pillar) including diffraction-limited finite-spot size correction:

At first glance, one might expect a more appreciable redshift from the strained regions, but as seen in Supplementary Figure 1, only a slight redshift in the exciton and trion peaks were resolved. However, this is not particularly surprising. It is worth mentioning that our excitation laser diffraction-limited spot-size has an FWHM of ~1 μm. This is important because the diameter of our pillars is only 150 nm. Within that area, the maximum strain is concentrated about 20 nm centered around the pillar's edge where the TMD bends over (Figure. 6a of the manuscript). Therefore, the spot size of the excitation laser is about two orders of magnitude larger than the strained region of interest. Due to this, the primary PL signal from the nano-pillar will still show a dominant unstrained neutral exciton peak. In contrast, the signal from the strained region becomes very dim in comparison. A similar result has been experimentally observed by Tyurnina et al.[14] when probing the strain in TMD nano-bubbles that are considerably smaller than the spot size of the laser.

To quantify this analysis and to see how dim the shifted strained excitons would be, we simulated the expected PL from the theoretical strain map given the spot-size correction. Simulation of the

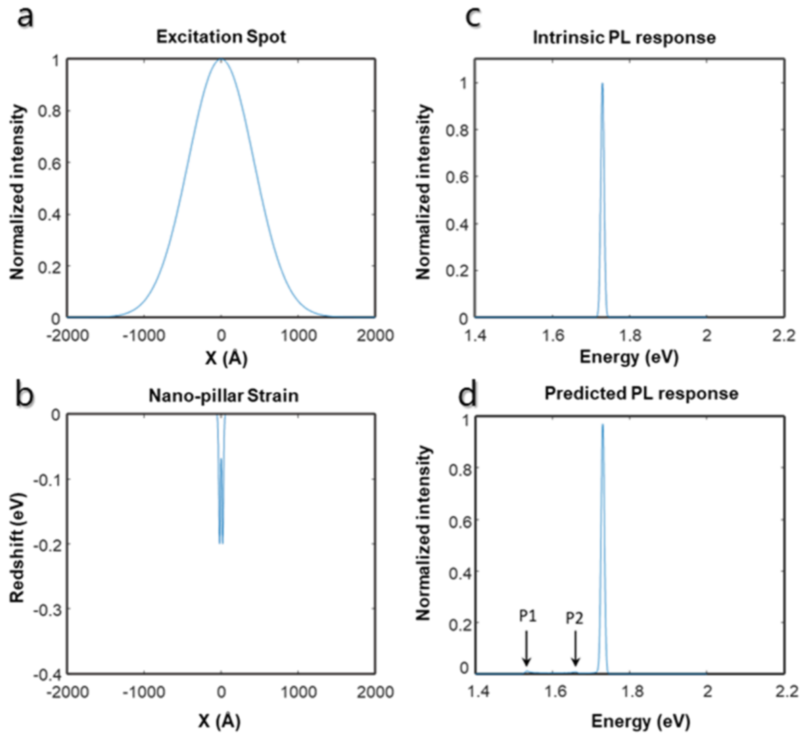

Supplementary Figure 2: Finite excitation spot size corrections. **(a)** Excitation spot with FWHM of ~1um. **(b)** Horizontal line cut of simulated strain profile of a 150 nm diameter nano-pillar. Note that line-cut travels through the center of the pillar. **(c)** Intrinsic PL response in unstrained regions obtained from fitting a gaussian with 10 meV FWHM to the experimental results. **(d)** Predicted PL response. The main observed peak is from the unstrained neutral exciton. Note that P2 and P1 correspond to the strained neutral exciton at nano-pillar positions. P1 is the peak corresponding to the maximum strain at the edge of the pillar and P1 peak is attributed to the top of the pillar where the strain relaxes to some extent.

strain profile over the nano-pillar shows that the maximum strain in the structure is concentrated at the pillar's edges where the 2D sheet and the nano-stressor meet. This can be approximated as two Gaussians with FWHM of 25 nm that are 50 nm apart, where the Gaussians' peak value is the maximum expected redshift (200 meV). Moreover, the spatial variation of excitation laser can also be represented as a Gaussian with FWHM of 1 μm. Since the experimental linewidth of the unstrained PL is ~10 meV, we can assume the PL response of each point under excitation is also a Gaussian with a linewidth of 10 meV. However, the Gaussian center frequency would correspond to the redshift of the PL due to strain at that position. This allows us to generate the total PL of the system under excitation by weighting the PL of each point in the system with the finite gaussian spot-size of the excitation laser. Furthermore, given that the lifetime of neutral excitons is in the picosecond range and the electric field due to strain gradient is negligible away from the pillar's edges, our funneling correction did not yield any considerable changes in the result.

Supplementary Figure 2 represents the simulated PL signal given that the gaussian spot-size of excitation laser directly shines on the strained region. Given that most of the light excites the unstrained area of TMD, the dominating signal is still the unstrained PL emission centered at 1.73 eV. The effect of the strain gradient is two very dim peaks (corresponding to the edge and the center of the pillar, respectively) redshifted from the unstrained PL signal as seen in Supplementary Figure 2. On a normalized PL, in the best-case scenario where the gaussian spot-size is centered precisely on the center of the nano-pillar, these peaks would correspond to about 1.15% and 0.85% intensity of the unstrained neutral exciton. Therefore as expected, the experimental PL spectra of the nano-pillar are dominated by the neutral unstrained excitons. Given that, in addition to observing a higher background emission corresponding to ~3% of the normalized PL at the lower energies, we also observed a strong charged exciton emission. We believe the strained neutral exciton peaks (P1, P2) are too dim to resolve within our diffraction-limited system and similar to nano-bubbles require a near-field imaging approach[15].

Finally, it is worth mentioning that the slightly redshifted exciton (1.8 meV) and Trion (3.1 meV) peaks were within the uncertainty of sample-to-sample variations and can be an indicator of a residual strain that occurs on a spatial scale that is comparable to the spot-size itself.

The primary PL signal's absolute redshift can be explained when the strained region is comparable to the spot-size (or when the funneling length is comparable to the spot-size, which is not the case for the neutral excitons given their picosecond lifetimes). Moreover, the broadening in the linewidth of the primary PL signal can be an indication of a linear strain gradient that exists on a similar scale to the spot-size. These two effects can be seen in Supplementary Figure 3, where the strain profile has a residual strain corresponding to at least 30 meV redshift everywhere under the spot-size, and in addition to that, there is a linear increase in the strain profile under the excitation field. This leads to a detected primarily PL signal that is both redshifted (due to residual strain) and broadened (due to strain gradient). Unfortunately, probing the strain via the broadening of the primary PL signal is also not straightforward, since in TMDs, with applied strain, due to reduction of phonon scattering from K-Q path, the intrinsic linewidth of WSe_2 also decreases (this effect is not considered in our analysis).

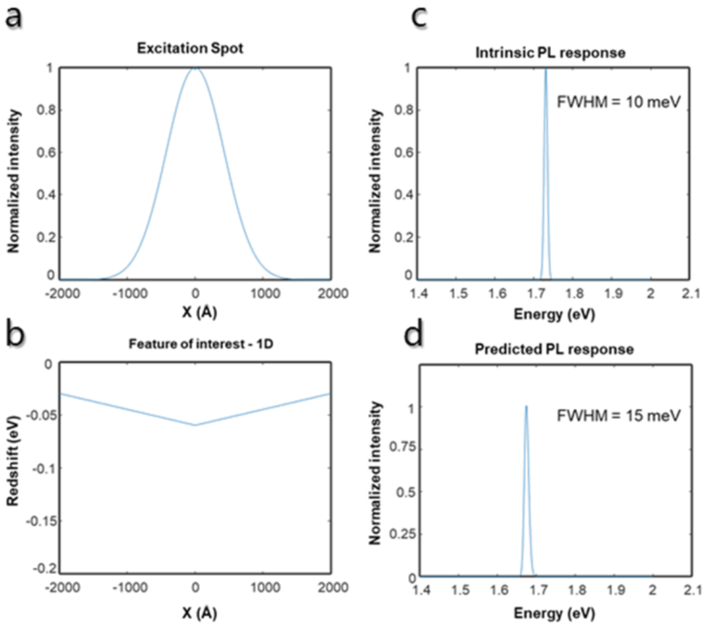

Supplementary Figure 3: Finite Excitation Spot Size Corrections for a linear strain profile. **(a)** Excitation spot with FWHM of ~1um. **(b)** Simulated strain profile that has a residual strain corresponding to a redshift of -0.03 eV which linearly increases to 0.06 eV at the center. **(c)** Intrinsic PL response in unstrained regions obtained from fitting a gaussian with 10 meV FWHM to the experimental results. **(d)** Predicted PL response. The PL signal is redshifted by 56 meV and its linewidth is broadened to 15 meV. The redshift is proportional to the average of strain per length, while the broadening corresponds to the linear slope of the strain profile.

Time resolved PL

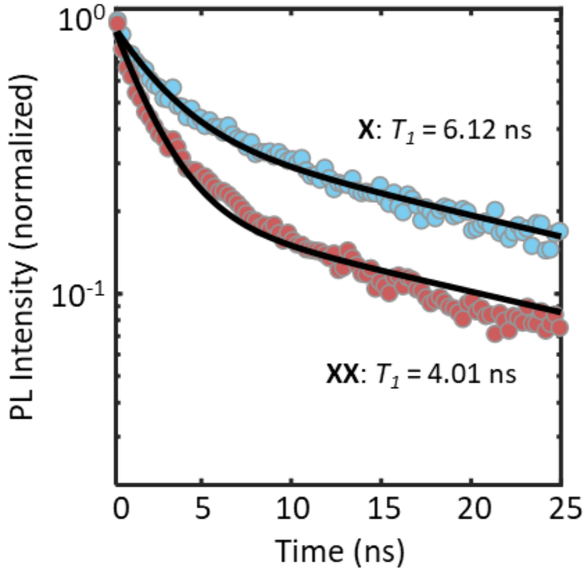

Supplementary Figure 4: Time-resolved photoluminescence of the exciton (X, blue) and biexciton (XX, red) acquired at 5 K. The data are fit with a biexponential function with fast decay times T_1 = 6.12 ns and T_1 = 4.01 ns, respectively. Comparable dynamics were observed for all biexciton-exciton pairs.

Spectral Diffusion of Exciton (X) and Biexciton (XX)

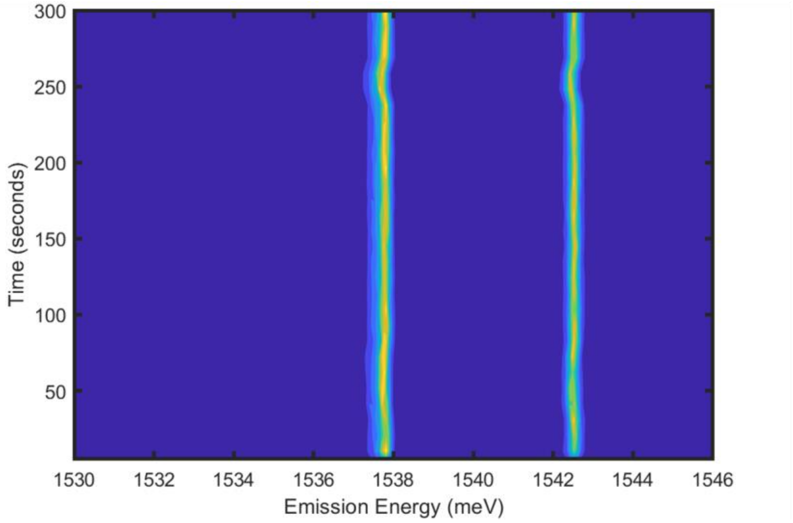

Supplementary Figure 5: Spectral Diffusion of Exciton (X) and Biexciton (XX). Micro-PL time series with 10 second integration time of each spectrum and 5 minute total integration time. Note that very little spectral diffusion is observed. However, the minor jitter seen at around t=250s is correlated for both X and XX emission lines, providing more evidence that these two emission lines are originating from the same emitter.

Supplementary Note 3: Temperature dependence of the single emitter properties

Temperature dependence of single-emitter properties. An exciting feature of defect-based emitters in atomically thin materials is strong confinement that makes them potentially less susceptible to thermal broadening and quenching at elevated temperatures compared to more conventional emitters, such as III-V self-assembled quantum dots. To examine thermal effects on the optical response, we first examine the peak energy shift with increasing temperature. We observe a red shift of the peak energy of ~5 meV at 150 K. The shift follows the bandgap variation according to the Varshni-form equation $E(T) = E(0) - \alpha T^2/(T + 170\text{ K})$, where α is associated with the relaxation of the lattice and 170 K is determined from experimental measurements of the Debye temperature. A fit to the data yields $\alpha = 5.3\times10^{-6}$ eV K^{-1}. This value is smaller than for native defects in WSe_2 grown with chemical vapor deposition, as expected. Here, the combination of intentional strain and deep chalcogen-based defects result in lower optical emission energies compared to native defects. The deeper confinement decouples the exciton energy from the temperature dependence of the lattice and bandgap leading to a smaller temperature-dependent shift in the energy.

The picture of deep defect states, which is supported by first-principles calculations discussed below, is also consistent with the temperature dependence of the PL peak intensity. In short, given a discrete energy state that lies within the semiconductor bandgap (with energy difference equal to $E_{confinement}$), to depopulate this state, a phonon has to supply the carrier with at least energy of $E_{confinement}$ to move the carriers out of the radiative state (Supplementary Figure 6. a). It is readily observable that as the confinement potential increases, the amount of phonon energy required to depopulate the emitter state would be higher. Therefore, the probability of this scattering would be lower. It is this exponential dependence of the Bose-Einstein population of phonons that translates to the exponential dependence of the quenching mechanism and the Arhenius equation $I(T) = I_0/(1 + R\exp(-E_A/kT))$ where R is equal to the ratio of the radiative (T_r) and non-

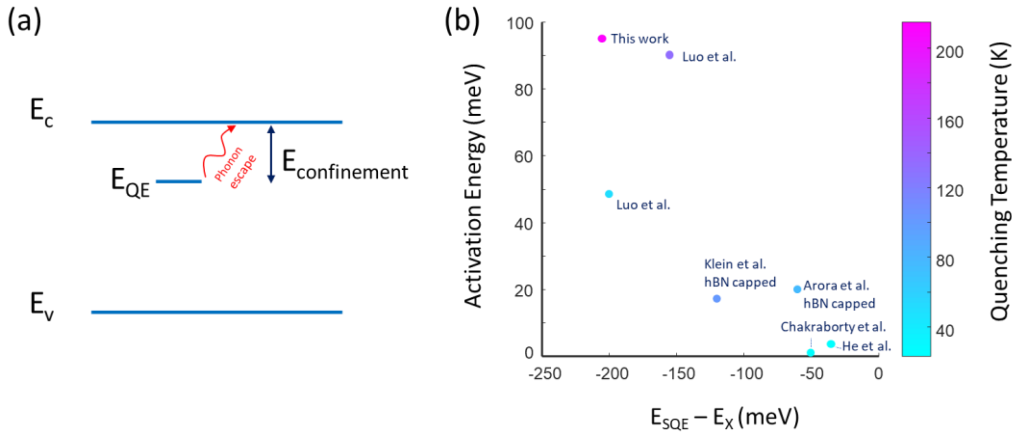

Supplementary Figure 6: Tempereature stability of TMD emitters (a) Phonon mediated depopulation of a discrete state. Confinement potential is defined as the energy difference between E_c and the emitting state. **(b)** Measured actiation energy, confinement potential (defined as the energy spacing between the single-photon emission line and the free exciton), and temperatures for WSe_2 emitters. Highest working temperature is this work. Chakraborty et al. refers to reference [2], He *et al.*[7], Luo *et al.* [13], Klein *et al.* [17], and Arora *et al.* [18].

radiative (T_{nr}) recombination lifetimes and E_A is the thermal activation energy that dissociates the defect-bound excitons.

The data are fit with $R = 19$ and $E_A = 95$ meV. This finding agrees with the small value of α. Assuming that the fast exciton recombination lifetime from the time-resolved PL is predominantly due to radiative emission, the measured value for R enables us to calculate the quantum yield $QY = T_r^{-1}/(T_r^{-1} + RT_r^{-1}) = 5$ %.

Here, we emphasize that activation energy (E_a) is not a fudge factor, and within this model, activation energy is directly correlated to $E_{confinement}$. Hence, similar to III-V quantum dot systems[16], it is expected that red-shifted emission lines persist at higher temperatures.To justify our point for the WSe_2 system, we have also compiled a 2D heatmap analysis of all studies[2,7,13,17,18] that have reported the temperature evolutions of their WSe_2 emitters. Again, we must note that, PL intensity is also sensitive to the coefficient preceding the exponential term in the Arhenius equation. This coefficient in simple models can be simplified to quantum efficiency, but in more rigorous models contains information about capture rates, capture cross-section, radiative and non-radiative lifetimes, and etc. Given that this term is material dependent and varies with the quality of material, this also introduces variability in the fitted range of activation energies. However, still, the trend of measured data in Supplementary Figure 6. b is as expected. Larger confinement potential (lower-emission energy) results in higher activation energy and higher thermal stability.

Finally, we examined the effects of exciton-phonon scattering on the homogeneous linewidth γ, which increases from ~60 μeV at 5 K to ~6 meV at 150 K. This behavior is indicative of thermally activated pure dephasing from exciton-phonon scattering of the form $\gamma(T) = \gamma_0 + aT + b\left(\exp(E_p/kT) - 1\right)^{-1}$, where the first term is the temperature independent linewidth, the second term corresponds to linear acoustic phonon-exciton scattering, and the last term corresponds to optical phonon-exciton scattering. A fit to the data yields $a = 15$ μeV/K and $E_p = 20$ meV. The measured acoustic phonon rate a is about a factor of four smaller than the free exciton in WSe_2, whereas the optical phonon energy E_p is nearly identical with the free exciton, suggesting that the phonon scattering mechanisms that dominate exciton-phonon broadening in pristine WSe_2 also play an important role for the quantum emitters.

These results provide a compelling route towards suppressing the quenching behavior of single emitters at room temperature. From the Arrhenius equation, it becomes clear that survival of the quantum emitter at higher temperatures is most sensitive to the parameter R associated with the quantum yield, which determines the point at which the emission is quenched, whereas the activation energy E_A determines the rate at which quenching occurs. Interesting, here we demonstrate that with low quantum yield but large E_A, the emitters survive up to a measured value of 150 K and a 1/e value of 215 K.

Statistics of single emitters across the ensemble

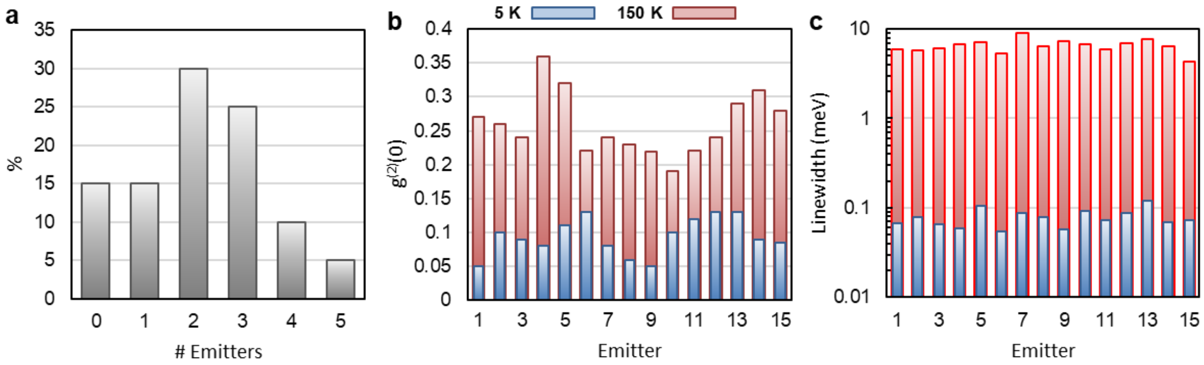

Supplementary Figure 7: Statistics of single emitters across the ensemble: **(a)** percentage of sites probed have N = 0-5 emitters, **(b)** $g^{(2)}(0)$ for 5 K (blue) and 150 K (red), and **(c)** homogeneous linewidth γ for 5 K (blue) and 150 K (red).

Supplementary Note 4: DFT Simulation details

Case of Single & Double Se vacancies:

Selenium vacancies are the most prevalent point defects in TMDs. Studies have shown that e-beam irradiation processes primarily contribute to the generation of chalcogen or double chalcogen vacancies due to their lower knock-off energy[19]. Moreover, the energy level of these states for both selenium (Supplementary Figure 8) and double selenium defects (Supplementary Figure 9) can be relatively close to E_c and move even closer with the application of strain. Because of these two factors, many initial studies attribute the defect bounded exciton to selenium vacancies. However, studies have shown that O_2 can easily be disassociated at Se vacancy sites in WSe_2[20]. It is also known that oxygen passivation moves the midgap Selenium defect states out of the bandgap[21] (reaffirmed in Supplementary Figure 10). Hence, the possibility of selenium vacancies giving rise to SPE seems low.

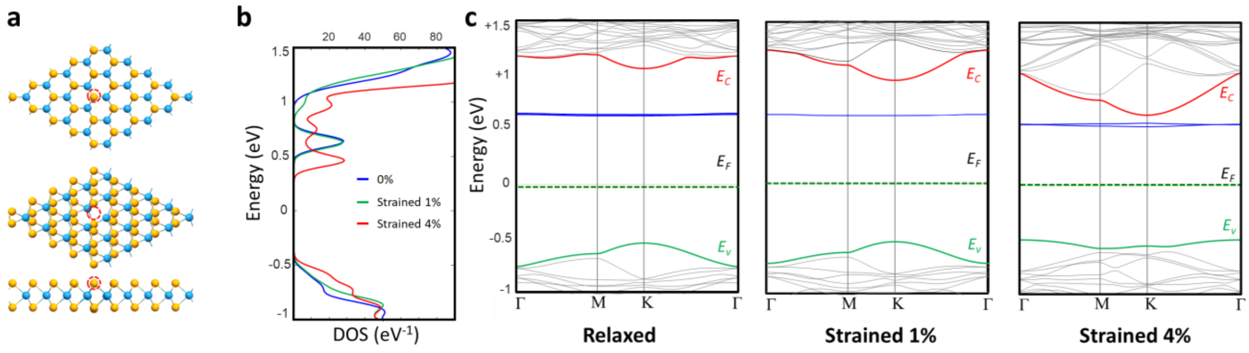

Supplementary Figure 8: Selenium vacancy **(a)** Relaxed atomic configuration of a single selenium vacancy. **(b)** Evolution of density of states with increased strain. **(c)** Evolution of the band diagram of selenium vacancy with strain.

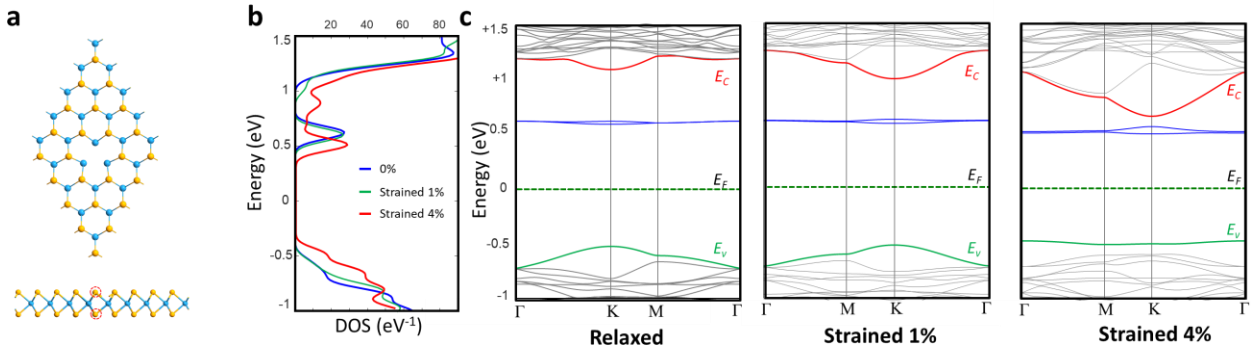

Supplementary Figure. 9: Double selenium vacancy **(a)** Relaxed atomic configuration of a double selenium vacancy. **(b)** Evolution of density of states with increased strain**. (c)** Evolution of the band diagram of the double selenium vacancy with strain.

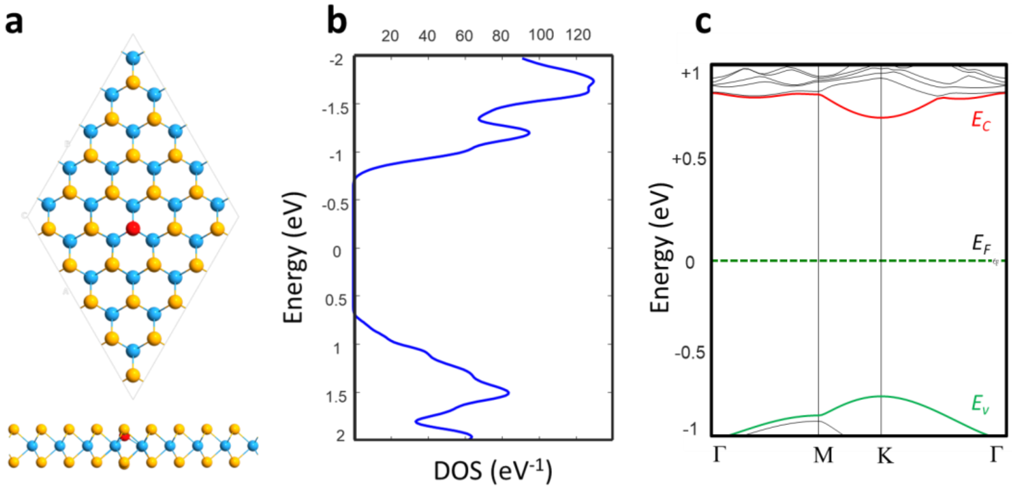

Supplementary Figure 10: Oxygen passivated selenium vacancy **(a)** Relaxed atomic configuration of a single selenium vacancy passivated by an oxygen atom. **(b)** Density of states. **(c)** Band diagram. Midgap states are now passivated and move to above/below conduction/valence band.

Case of Tungsten Centered Vacancies:

In an initial STM study, it was observed that dominant vacancies in WSe_2 are centered around Tungsten vacancies[22]. This was a favorable result since not only can tungsten vacancies provide energy levels close to E_c (at relatively high strain), but also other energy levels close to E_v, which could explain the unusual ambipolar and p-type behavior of WSe_2 (Supplementary Figure 11). However, considering new studies[20], it is evident that the prevalence of either Se or W vacancies cannot be generalized for every WSe_2 at this time and will be dependent on their growth conditions. While the morphology of dominant defect types in WSe_2 is under debate, however, studies have shown that under e-beam irradiation, for higher dose or exposure time, pore vacancies (lost metal with its chalcogens) can occur in TMDs[23,24]. In this process, prolonged e-beam exposure leads to

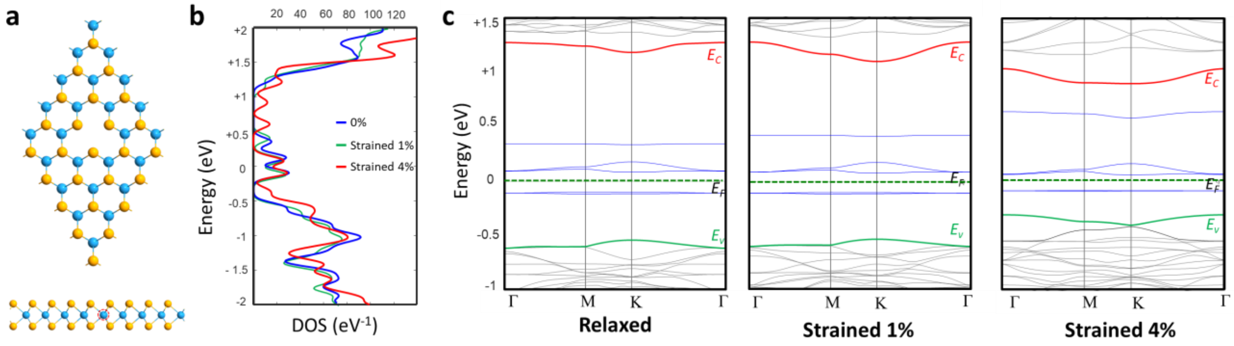

Supplementary Figure 11: (a) Relaxed atomic configuration of a single tungsten vacancy. **(b)** Evolution of density of states with increased strain. **(c)** Evolution of the band diagram of the tungsten vacancy with strain.

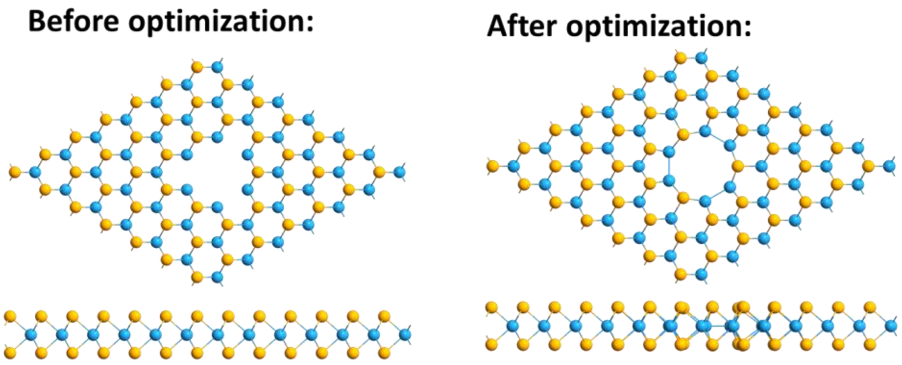

Supplementary Figure 12: WSe_6 geometry relaxation (a) Atomic structure of WSe_2 after removing a WSe_6 complex **(b)** Formation of a nano-pore upon bond rotations and restructuring of surrounding atoms during geometry relaxation.

the destabilization of the transition metal bond, which causes the transition metal to migrate away from the site. Afterward, chalcogen atoms connected to the migrated transition metal tend to disassociate very easily[25]. Hence instead of a single Tungsten vacancy, defects complexes such as WSe_3 or WSe_6 are formed. These defect sites then relax to more stable geometries through bond rotation and restructuring that resemble nano-pores, as seen in Supplementary Figure 12 (hence the term pore vacancy).

Another observed vacancy complex in WSe_2 under e-beam irradiation is the trefoil defects. Excess selenium divacancies created during e-beam irradiation can migrate and form trefoil-like structures through bond rotations[26]. These defects were also observed in our simulations where three chalcogen divacancies were first created in each other's proximity, and after geometry relaxation, the final structure relaxed to a trefoil-like defect structure similar to experimental observations as seen in Supplementary Figure 13. a. However, due to the relatively large size of these defects, they introduce many states in the midgap, which can substantially increase non-radiative decay probability, which makes them a less compelling candidate.

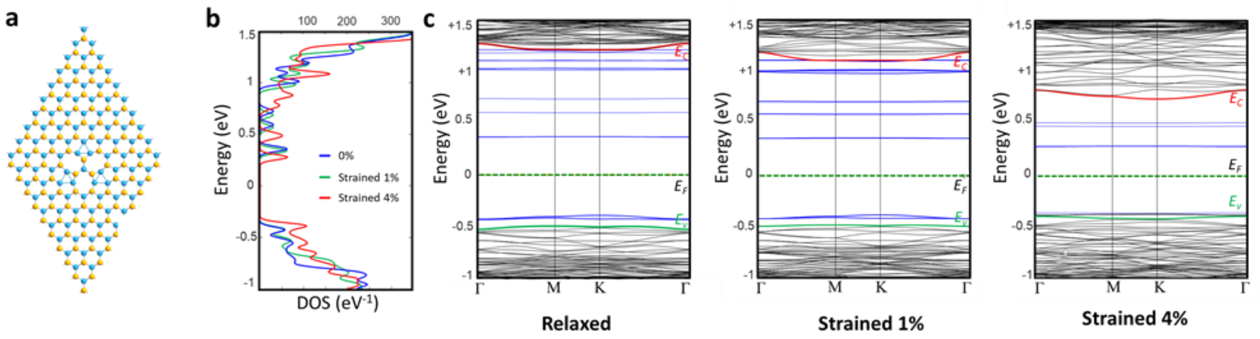

Supplementary Figure 13: WSe_6 complex (a) Relaxed atomic configuration of the trefoil defect. **(b)** Evolution of density of states with increased strain. **(c)** Evolution of the band diagram of the trefoil defect with strain.

References

1. Srivastava, A. *et al.* Optically active quantum dots in monolayer WSe_2 . *Nat. Nanotechnol.* **10**, 491–496 (2015).
2. Chakraborty, C., Kinnischtzke, L., Goodfellow, K. M., Beams, R. & Vamivakas, A. N. Voltage-controlled quantum light from an atomically thin semiconductor. *Nat. Nanotechnol.* **10**, 507–511 (2015).
3. Kumar, S., Kaczmarczyk, A. & Gerardot, B. D. Strain-induced spatial and spectral isolation of quantum emitters in mono- and bilayer WSe_2. *Nano Lett.* **15,** 7567–7573 (2015).
4. Tonndorf, P. *et al.* Single-photon emission from localized excitons in an atomically thin semiconductor. *Optica* **2**, 347-352 (2015).
5. He, Y.-M. *et al.* Single quantum emitters in monolayer semiconductors. *Nat. Nanotechnol.* **10**, 497–502 (2015).
6. Kern, J. et al. Nanoscale positioning of single-photon emitters in atomically thin WSe_2. *Adv. Mater.* **28,** 7101–7105 (2016).
7. He, Y. M. *et al.* Cascaded emission of single photons from the biexciton in monolayered WSe_2. *Nat. Commun.* **7**, 13409 (2016).
8. Branny, A., Kumar, S., Proux, R. & Gerardot, B. D. Deterministic strain-induced arrays of quantum emitters in a two-dimensional semiconductor. *Nat. Commun.* **8**, 15053 (2017).
9. Palacios-Berraquero, C. *et al.* Large-scale quantum-emitter arrays in atomically thin semiconductors. *Nat. Commun.* **8**, 15093 (2017).
10. Luo, Y. et al. Deterministic coupling of site-controlled quantum emitters in monolayer WSe_2 to plasmonic nanocavities. *Nat. Nanotechnol.* **13,** 1137–1142 (2018).
11. Rosenberger, M. R. *et al.* Quantum Calligraphy: writing single-photon emitters in a two-dimensional materials platform. *ACS Nano* **13**, 904–912 (2019).
12. Kim, H., Moon, J. S., Noh, G., Lee, J. & Kim, J. H. Position and frequency control of strain-induced quantum emitters in WSe_2 monolayers. *Nano Lett.* **19**, 7534–7539 (2019).
13. Luo, Y., Liu, N., Li, X., Hone, J. C. & Strauf, S. Single photon emission in WSe_2 up 160 K by quantum yield control. *2D Mater.* **6**, 035017 (2019).
14. Tyurnina, A. V. *et al.* Strained bubbles in van der Waals heterostructures as local emitters of photoluminescence with adjustable wavelength. *ACS Photonics* **6**, 516–524 (2019).
15. Darlington, T. P. *et al.* Imaging strain-localized excitons in nanoscale bubbles of monolayer WSe_2 at room temperature. *Nat. Nanotechnol.* **15**, 854–860 (2020).
16. Le Ru, E. C., Fack, J. & Murray, R. Temperature and excitation density dependence of the photoluminescence from annealed InAs/GaAs quantum dots. *Phys. Rev. B* ***67***, 245318 (2003).
17. Klein, J. *et al.* Site-selectively generated photon emitters in monolayer MoS_2 via local helium ion irradiation. *Nat. Commun.* **10**, 1–8 (2019). Arora, A. *et al.* Dark trions govern the temperature-dependent optical absorption and emission of doped atomically thin semiconductors. *Phys. Rev. B* **101**, 241413 (2020).
18. Komsa, H. P. *et al.* Two-dimensional transition metal dichalcogenides under electron irradiation: defect production and doping. *Phys. Rev. Lett.* **109**, 035503 (2012).
19. Zheng, Y. J. *et al.* Point defects and localized excitons in 2D WSe_2. *ACS Nano* **13**, 6050–6059 (2019).

20. Lu, J. *et al.* Atomic healing of defects in transition metal dichalcogenides. *Nano Lett.* **15**, 3524–3532 (2015).
21. Zhang, S. *et al.* Defect structure of localized excitons in a WSe_2 monolayer. *Phys. Rev. Lett.* **119**, 046101 (2017).
22. Wang, S. *et al.* Atomic structure and formation mechanism of sub-nanometer pores in 2D monolayer MoS_2. *Nanoscale* **9**, 6417–6426 (2017).
23. Ryu, G. H. *et al.* Atomic structure and dynamics of self-limiting sub-nanometer pores in monolayer WS_2. *ACS Nano* **12**, 11638–11647 (2018).
24. Zhou, W. *et al.* Intrinsic structural defects in monolayer molybdenum disulfide. *Nano Lett.* **13**, 2615–2622 (2013).
25. Lin, Y. C. *et al.* Three-fold rotational defects in two-dimensional transition metal dichalcogenides. *Nat. Commun.* **6**, 6736 (2015).
